# Structure–Biological Function Relationship Extended to Mitotic Arrest-Deficient 2-Like Protein Mad2 Native and Mutants-New Opportunity for Genetic Disorder Control

**DOI:** 10.3390/ijms151121381

**Published:** 2014-11-18

**Authors:** Speranta Avram, Adina Milac, Maria Mernea, Dan Mihailescu, Mihai V. Putz, Catalin Buiu

**Affiliations:** 1 Department of Anatomy, Animal Physiology and Biophysics, Faculty of Biology, University of Bucharest, 91-95 Spl. Independentei, Bucharest 050095, Romania; E-Mails: speranta.avram@gmail.com (S.A.); amilac@biochim.ro (A.M.); dan.mihailescu@bio.unibuc.ro (D.M.); catalin.buiu@acse.pub.ro (C.B.); 2 Department of Bioinformatics and Structural Biochemistry, Institute of Biochemistry of the Romanian Academy Bucharest (IBAR), 296 Spl. Independentei, Bucharest 060031, Romania; 3 Department of Biology-Chemistry, Faculty of Chemistry, Biology, Geography, West University of Timisoara, 16th Pestalozzi Str., Timisoara 300115, Romania; E-Mail: mv_putz@yahoo.com; 4 Department of Automatic Control and Systems Engineering, Politehnica University of Bucharest, 313 Spl. Independentei, Bucharest 060042, Romania

**Keywords:** chromosomal instability prediction, mitotic arrest-deficient protein Mad2, computational mutagenesis, quantitative structure-activity relationship (QSAR)

## Abstract

Overexpression of mitotic arrest-deficient proteins Mad1 and Mad2, two components of spindle assembly checkpoint, is a risk factor for chromosomal instability (CIN) and a trigger of many genetic disorders. Mad2 transition from inactive open (O-Mad2) to active closed (C-Mad2) conformations or Mad2 binding to specific partners (cell-division cycle protein 20 (Cdc20) or Mad1) were targets of previous pharmacogenomics studies. Here, Mad2 binding to Cdc20 and the interconversion rate from open to closed Mad2 were predicted and the molecular features with a critical contribution to these processes were determined by extending the quantitative structure-activity relationship (QSAR) method to large-size proteins such as Mad2. QSAR models were built based on available published data on 23 Mad2 mutants inducing CIN-related functional changes. The most relevant descriptors identified for predicting Mad2 native and mutants action mechanism and their involvement in genetic disorders are the steric (van der Waals area and solvent accessible area and their subdivided) and energetic van der Waals energy descriptors. The reliability of our QSAR models is indicated by significant values of statistical coefficients: Cross-validated correlation *q*^2^ (0.53–0.65) and fitted correlation *r*^2^ (0.82–0.90). Moreover, based on established QSAR equations, we rationally design and analyze nine *de novo* Mad2 mutants as possible promoters of CIN.

## 1. Introduction

Mitotic cell division is the process in which a single chromosome is replicated into two identical copies named sister chromatids [1]. Further, during the bi-orientation process, the sister chromatids are attached to microtubules from the two opposite spindle poles by specific protein assemblies named kinetochores [2,3]. Usually, the correct chromosome segregation is ensured by the activation of an intercellular correction mechanism called mitotic spindle assemble checkpoint (SAC) which delays the onset of anaphase until all sister chromatids are paired correctly [3,4]. SAC components, represented by mitotic arrest-deficient proteins (Mad1, Mad2 and Mad3), protein kinases (Bub1, BubR1, Bub3, TAO1), Mps1 protein kinase and kinetochore proteins Rod and ZW10, interact in order to inactivate another huge protein complex, namely the anaphase-promoting complex (APC). APC is inactivated until all sister chromatids are properly attached to spindle microtubules [5,6]. APC inhibition requires a direct interaction of mitotic arrest-deficient protein Mad2 with cell-division cycle protein 20 (Cdc20), an important member of APC, and it was shown that for the effective interaction of Mad2 with Cdc20, the presence of mitotic arrest-deficient protein Mad1 is critical [6,7]. SAC functioning requires the activation of mitotic checkpoint complex (MCC), the interaction of activated Mad2 with its specific ligands Mad1 and/or Cdc20 and the inhibition of Cdc20 trough phosphorylation events that involve kinases in interaction with Mad2 [8]. Biochemically, the molecular events which lead to SAC-APC interaction are: (i) First, the Mad1–Mad2 core complex catalyzes the conformational activation of the two-state protein Mad2, namely the transition from inactive open-Mad2 to active closed-Mad2 [9,10]; and (ii) second, closed-Mad2 in the Mad1–Mad2 complex is released and binds to Cdc20. Furthermore, Mad2–Cdc20 complex promotes the binding of BubR1-Bub3 to Cdc20, forming the anaphase-promoting complex/cyclosome (APC/C) inhibitory mitotic checkpoint complex [11,12].

Structurally, Mad2 comprises 205 amino acids [13], presents specific phosphorylation sites [12,13] and has two native conformations: The inactive open conformer (O-Mad2) and the active closed conformer (C-Mad2) [4]. C-Mad2 conformer is adopted upon binding to Cdc20 or to Mad1 [14,15,16] and it is more active than O-Mad2 conformer during the interaction with Cdc20 [17]. It was shown that Mad1–Mad2 core complex involves Mad2 as a dimer, while Mad2–Cdc20 core complex formation requires Mad2 as a monomer [10]. Recent studies identified the *C*-terminal region as the most active Mad2 domain and showed that its deletion induces a significant alteration of Mad2 function [14]. The importance of Mad2 *C*-terminal region and its involvement in Mad2 conformational transition was shown by the deletion of the 10 *C*-terminal residues in Mad2, which arrested the protein in the open conformation and made it unable to interact to either Mad1 or Cdc20 [17].

Structure-based mutagenesis studies indicated that: (i) Certain pairs of amino acid substitutions (R133A, F186A; R133A, T188A; R133A, H191A; R133A, V197A or R133A, Y199A) in Mad2-*C*-terminal domain are associated with Mad2 folding only in open conformation which leads to the failure of Mad2 binding to Cdc20 and indicates the importance of Mad2-*C*-terminal domain integrity for Mad2 function [10]; (ii) other pairs of amino acid substitutions (R133A, L13A; R133A, L153A; R133A, Y156A; R133A, W75A) are associated with Mad2 folding only in closed conformation [10]; (iii) Mad2 R133E, Q134A mutants are unable to recruit a second Mad2 molecule to form Mad2 dimers and fail to inhibit APC/C [18]; and (iv) other interesting Mad2 residues whose substitution abolishes the affinity of Mad2 for the specific ligands Mad1 and Cdc20 or induce critical Mad2 structural folding defects are L13A, W75A, L153A, Y156A, F186A, T188A, H191A, and V197A [10].

The Mad2 dimerization process was also studied in the case of native and mutant Mad2 proteins. In the case of Mad2 RQ mutants, such as those bearing R133E and Q134A substitutions, the dimerization process is impaired, leading to a loss of APC/C inhibition during chromosomal amplification [16,18]. Mad2 protein bearing the R133A substitution is able to adopt both O-Mad2 and C-Mad2 conformation. Unlike O-Mad2 R133A, monomeric C-Mad2 R133A is able to inhibit APC/C. O-Mad2 and C-Mad2 can form dimers, but the resulting asymmetric O-C dimer is less active in APC/C inhibition [17]. Other mutations that disrupt Mad2 dimerization involve residues found at the dimerization interface: R133, Q134, T140, and F141 [10]. In the dimer structure, these residues form hydrogen bonds or interact through electrostatic forces with residues from the neighbouring monomer, explaining why C-Mad2 is the only conformer that has the ability to form dimers [10].

Clinical studies have shown that the variation in Mad2 expression is associated with abnormal chromosome numbers or chromosome instability [19,20,21,22,23].

Encouraged by preclinical and clinical data, by the reduced number of Mad2 computational studies and the lack of quantitative structure-activity relationship (QSAR) studies, here we take an original approach and extend for the first time, the applicability of QSAR methods on large molecules like native Mad2 and its mutants, in order to determine a possible correlation between Mad2 family features and their mechanism of action during chromosome segregation and/or their involvement in chromosome instability. Our previously successful application of QSAR/SAR approach to predict activity of large proteins such as glycoprotein HIV-1 gp120 [24] or small proteins such as peptides from the mastoparan family [25,26,27] allowed us to identify molecular descriptors that are critical for biological activity, such as hydrophobicity (e.g., the ratio of concentrations of a compound between the two solutions usually considered as water-octanol), steric (e.g., molecular surface areas) or count of atoms and bonds types (e.g., polar and hydrophobic atoms, rigid and rotatable bonds).

Usually, QSAR methods are applied to predict the biological activity of small molecules like drugs, but here we successfully used the methods to correlate the predicted and experimental features of Mad2 proteins family expressed as: (i) Binding to Cdc20; and (ii) the interconversion folding rate from inactive O-Mad2 to active C-Mad2 configurations.

When QSAR models are employed to accurately predict the features of proteins, an appropriate selection of molecular descriptors encoding these proteins features is critical. Here, we establish that the molecular descriptors like van der Waals energy, van de Waals surface and water accessible surface areas corresponding to hydrophobic atoms, and count of rigid and rotatable bonds, are critical for the formation of Mad2–Cdc20 core complex, while hydrophobic and electronic dipole moments, van der Waals surface areas corresponding to polar and hydrogen bond acceptor atoms, are critical for the folding of Mad2 conformers. Considering that Mad2 family is widely involved in many types of genetic diseases, the rational-design and also the prediction of chromosome instability induced by putative *de novo* Mad2 mutants in humans is essential in preclinical and clinical studies. Because sometimes these studies are difficult to perform, here we used computational mutagenesis methods for rational design of nine *de novo* Mad2 mutants and we applied QSAR methods to predict their possible features such as the Cdc20 binding and the interconversion rate between open and closed conformations.

To gain a deeper understanding of the mechanism by which experimentally identified Mad2 mutants [10] are involved in chromosome instability, we extended the structural study of mutant Mad2 proteins by analyzing the fluctuation of molecular descriptors determined strictly for the active domain of Mad2 represented by its *C*-terminal domain.

In the present study, we use computational methods to generate accurate QSAR models on cellular proteins thus opening new perspectives for understanding the tumorigenesis mechanism and the implication of mitotic spindle assemble check point proteins in genetic diseases.

## 2. Results and Discussion

### 2.1. Results

#### 2.1.1. QSAR Models Predicted Mad2 Native and Mutants Binding to Cdc20

We generated multiple QSAR models to predict binding of Mad2 to Cdc20. In the initial QSAR models, a huge number of descriptors was calculated, but during model validation most descriptors were shown to have insignificant contributions to the correlation between experimental and predicted binding affinities and were excluded from the models. In QSAR models 1 and 2 were selected only those combinations of descriptors (van E-vdW, vsa_hyd, ASA_hyd and b_rigid_ and b_rot_ bonds) showing a clear improvement of statistical coefficients: *q*^2^-cross-validated correlation coefficient equal to 0.53/0.65; *r*^2^ fitted correlation coefficient equal to 0.82/0.83. All statistical coefficients obtained for QSAR models are presented in Table 1.

The predictive power of QSAR models 1 and 2 was assessed by predicting p*K*_dCdc20_ values (the common logarithm of inverse binding affinities) for testing set molecules. The predicted binding of Mad2 native and mutants with Cdc20 in training and testing sets were calculated according to the QSAR equations generated and presented in Table 2. The obtained values were compared with experimental Mad2 protein activities. Also the residual values expressed as the difference between experimental and predicted Mad2 activity are shown.

Also, experimental studies [10] demonstrated that the same Mad2 mutants (R133A/P164A; R133A/T187A; R133A/K192A) are able to adopt both open and closed configurations and are able to interact with Cdc20, but the affinities were not determined. The power of our QSAR models to predict the binding of Mad2 mutants with Cdc20 was applied for these mutants.

**Table 1:** Summary of quantitative structure-activity relationship (QSAR) statistical parameters for QSAR models 1–3: *q*^2^ (cross-validated *r*^2^), fitted correlation *r*^2^, root mean square error (RMSE), cross-validated RMSE and Fisher (*F*) test.

QSAR Model	*q*^2^	*r*^2^	RMSE	Cross-Validated RMSE	*F* test
QSAR model 1	0.53	0.82	0.15	0.27	13.22
QSAR model 2	0.65	0.83	0.14	0.20	10.03
QSAR model 3	0.60	0.90	0.10	0.25	10.23

**Table 2:** Experimental and predicted p*K*_dCdc20_ (the common logarithm of inverse binding affinities) and pCR (the common logarithm of inverse conversion rates) of Mad2 native and mutants and residual values obtained by applying QSAR models 1–3 (in brackets). Bold numbers indicate values for test sets and italics indicate predicted values for which corresponding activities were not detected experimentally.

Mad2 Mutant	QSAR Model 1	QSAR Model 2	QSAR Model 3	pCR_exp_	p*K*_(dCdc20)exp_	*K*_d_ (µM)
	p*K*_(dCdc20)pred_	p*K*_(dCdc20)pred_	pCR_pred_			
R133A/L84A	6.92 (−0.12)	7.00 (−0.20)	**4.33 (−0.41)**	3.92	6.80	0.16
R133A/I88A	6.52 (0.18)	6.58 (0.12)	**4.14 (0.62)**	4.76	6.70	0.20
R133A/F151A	6.52 (0.01)	6.61 (−0.08)	**4.25 (0.75)**	5.00	6.53	0.29
R133A/L154A	5.93 (−0.02)	5.97 (−0.06)	4.29 (0.08)	4.37	5.91	1.21
R133A/D158A	6.83 (0.05)	6.90 (−0.02)	4.17 (0.13)	4.30	6.88	0.13
R133A/V163A	6.58 (0.05)	6.63 (0.00)	4.20 (−0.02)	4.18	6.63	0.23
R133A/S170A	6.87 (0.22)	6.95 (0.14)	**3.84 (0.47)**	4.31	7.09	0.081
R133A/E179A	6.86 (−0.07)	6.88 (−0.09 )	4.32 (−0.02)	4.30	6.79	0.16
R133A/V181A	6.19 (−0.19)	6.27 (−0.27)	4.27 (0.15)	4.42	6.00	0.10
R133A/K200A	6.81 (−0.05)	6.82 (−0.06)	4.56 (−0.01)	4.55	6.76	0.17
R133A/L13A	6.71 (0.21)	6.79 (0.13)	Eq. not applied	NA	6.92	0.12
Native	6.98 (0.02)	7.05 (−0.05)	*5.76*	NA *	7.00	0.1
R133A	**7.66 (−0.81)**	**7.76 (−0.91)**	4.30 (0.00)	4.30	6.85	0.14
R133A/L153A	**7.19 (−0.52)**	**7.25 (−0.58)**	Eq. not applied	NA	6.67	0.21
R133A/D160A	**7.23 (−0.65)**	**7.32 (−0.74)**	4.07 (−0.07)	4.00	6.58	0.26
R133A/Y156A	**6.38 (−0.13)**	**6.48 (−0.23)**	Eq. not applied	NA	6.25	0.56
R133A/T12A	**5.94 (1.01)**	**5.98 (0.97)**	3.55 (0.10)	3.65	6.95	0.11
L13A	*6.96*	*7.01*	Eq. not applied	NA *	ND	ND
R133A/P164A	*6.53*	*6.62*	4.32 (−0.27)	4.05	ND	ND
R133A/T187A	*6.60*	*6.69*	3.67 (−0.11)	3.56	ND	ND
R133A/K192A	*6.53*	*6.53*	*3.30*	ND	ND	ND
R133A/W167A	Eq. not applied	Eq. not applied	**4.09 (−0.40)**	3.96	NBD	NBD

ND = binding was not determined experimentally; NBD = no binding was detected experimentally; NA = not applicable; NA * = there are no experimental data; Eq. not applied = the QSAR equation was not applied due to the lack of experimental data required for comparison with predicted p*K*_dCdc20_ or pCR.

**Figure 1:**
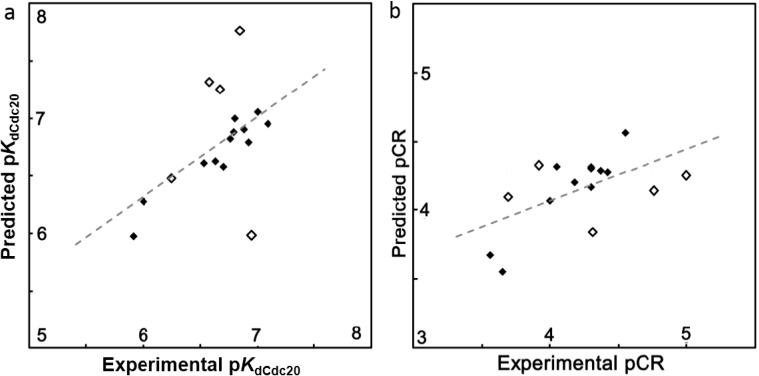
(**a**) Correlation between experimental and predicted binding of Mad2 native and mutants (p*K*_dCdc20_) obtained by QSAR model 2 (*q*^2^ = 0.65, *r*^2^ = 0.83); and (**b**) correlation between experimental and predicted conversion rate of Mad2 native and mutants (pCR) obtained by QSAR model 3 (*q*^2^ = 0.60, *r*^2^ = 0.90). Black dots correspond to molecules in the training set and white (open) dots to molecules in the test set.

Data shown in Table 2 is supported by the appropriate correlations between experimental and predicted binding of Mad2 native and mutants to Cdc20 (p*K*_dCdc20_ of Mad2) when van der Waals energy, subdivided van der Waals and water accessible surface areas induced by hydrophobic atoms and count of rigid and rotatable bonds descriptors (QSAR model 2) were simultaneously considered (Figure 1a). The good quality of our statistical parameters *q*^2^ and *r*^2^ is supported by a reasonable distribution of scatter in Figure 1a.

Chromosome instability disorders are associated with mutations in proteins from SAC complex. We previously mentioned the deep involvement of Mad2 mutations in different types of cancer and other genetic disorders. Thus, rational design of new Mad2 mutants with or without CIN inductor effect would be a major breakthrough in the pharmacogenomic research field. With this aim, we created a set of nine *de novo* Mad2 mutants with single substitutions at residue F186 and double substitutions at R133 and F186, for which we predicted Cdc20 binding affinities and compared with values from parent template and native structures. Results based on predictive ability of 3D-QSAR models 1 and 2 show that parent template Mad2 F186A and Mad2 R133A/F186A, and two *de novo* mutants R133A/F186S and R133A/F186N present theoretical p*K*_dCdc20_ values smaller (5.34–5.95) than those determined for Mad2 native and Mad2 mutants able to bind Cdc20. Further, four of *de novo* Mad2 mutants have predicted p*K*_dCdc20_ values comparable (6.22–6.67) with those of Mad2 mutants. Predicted p*K*_dCdc20 _of three *de novo* Mad2 mutants (F186M, F186W, R133A/F186 M) appear be higher than p*K*_dCdc20_ of Mad2 mutants (Table 3). For a few *de novo* mutants we extended our QSAR model 3 predicted power (see Discussion Section).

**Table 3:** Predicted p*K*_dCdc20_ of *de novo* Mad2 mutants and their templates by applying QSAR models 1 and 2.

Mad2 Mutants	QSAR Model 1	QSAR Model 2	p*K*_(dCdc20)exp_
	p*K*_(dCdc20)pred_	p*K*_(dCdc20)pred_	
	**Templates**		
F186A	5.77	5.96	NBD
R133A/F186A	5.34	5.44	NBD
	***de novo***** Mad2 Mutants**		
F186M	7.37	7.44	ND *
F186S	6.22	6.30	ND *
F186T	6.67	6.71	ND *
F186W	7.40	7.46	ND *
F186N	6.40	6.51	ND *
R133A/F186M	7.11	7.24	ND *
R133A/F186S	5.92	6.01	ND *
R133A/F186T	6.30	6.40	ND *
R133A/F186N	5.64	5.73	ND *

NBD = no binding was detected experimentally; ND * = binding was not determined experimentally as no experiments were performed on these mutants.

#### 2.1.2. QSAR Model Predicted Mad2 Native and Mutants Function Expressed as O-Mad2–C-Mad2 Interconversion Rate

Based on experimental data [10] demonstrating the importance of the conversion rate from open (inactive) to closed (active) conformations of Mad2 protein for Cdc20 binding, we generated a QSAR model that predicts the rate of conversion of Mad2 native and mutants using the observed molecular descriptors deeply involved in this process. Similarly to the QSAR models presented before, for validation of the third QSAR model, we initially considered a large number of descriptors with very poor values of statistical parameters. By excluding the non-relevant molecular descriptors, we obtained a set of four descriptors that produce a significant improvement of the statistical coefficients (*q*^2^ = 0.60, *r*^2^ = 0.90) (Table 1). These descriptors are hydrophobic and electronic dipole moments, and subdivided van der Waals surface areas induced by polar (vsa_pol) and hydrogen bond acceptor (vsa_acc) atoms. The experimental and predicted open–closed-Mad2 conversion rates in the training and test sets (in bold) are presented in Table 2. Also Table 2 shows the residual activity expressed as the difference between predicted and experimental values (in brackets). Because previous studies [10] did not detect the open–closed Mad2 conversion rate for native and Mad2 R133A/K192A mutant, but mentioned that both Mad2 proteins undergo conformational changes, we applied the predictive power of QSAR model 3 to evaluate the rate of conformational conversion for these two structures. Correlations between the predicted and experimental conversion rates are illustrated in Figure 1b. The good statistic values of *q*^2^ and *r*^2^ parameters are supported by reasonable scattering represented in Figure 1b.

#### 2.1.3. Structure–Function Relationship Model of Mad2 Native and Mutants at *C*-Terminal Domain Residues

**Figure 2:**
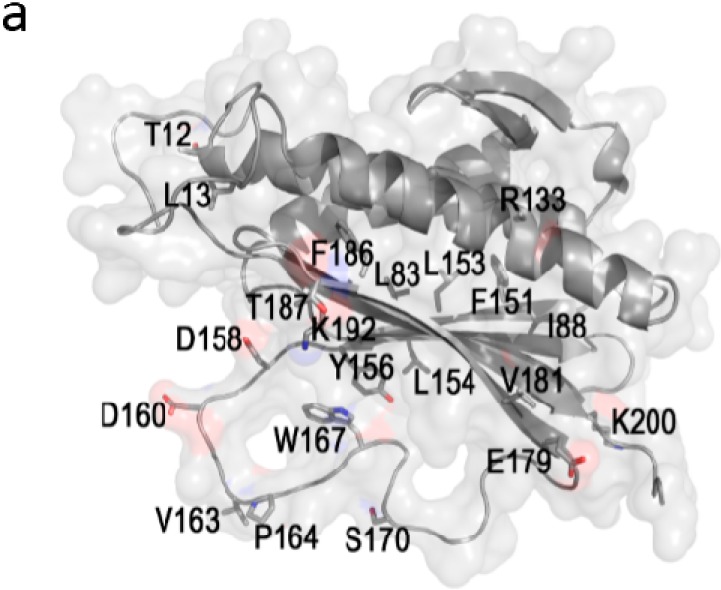

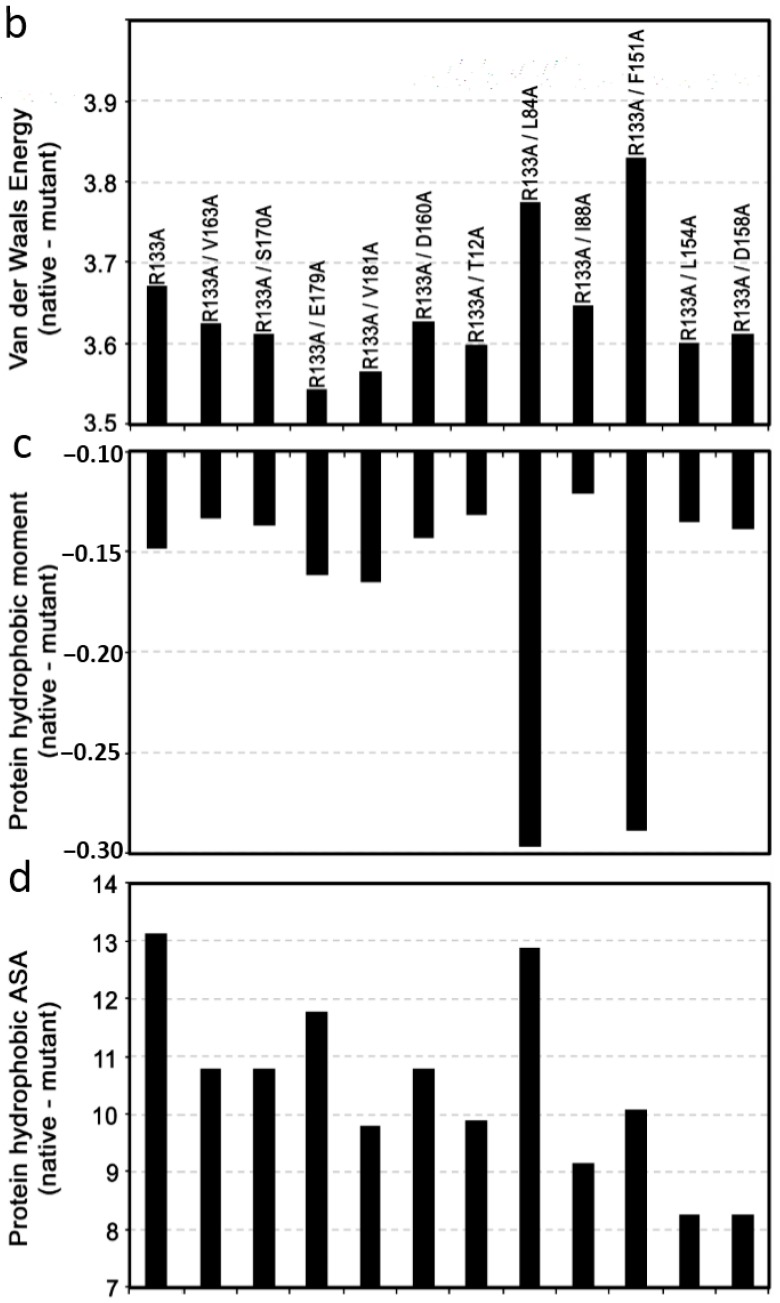
Variation of molecular descriptors for most relevant mutants. (**a**) Mad2 structure (Protein Data Bank code: 1S2H [17]) in ribbon representation, molecular surface colored light gray and mutants side chains represented as sticks. Only side chains of most relevant mutants are displayed, to illustrate their position within the structure; and (**b**–**d**) Deviation of molecular descriptors calculated as the difference between values for native protein and mutants: Van der Waals energy (**b**), protein hydrophobic moment (**c**) and protein hydrophobic ASA (**d**). Order of mutants (thus also mutants labelling) is the same in all plots.

Based on clinical and structural data [10,15] demonstrating that Mad2 *C*-terminal active domain is deeply involved in the interactions with specific partners (Mad1 and Cdc20) and undergoes major conformational changes to allow these interactions, we evaluated the molecular descriptors values of C-Mad2 native and mutants, including *de novo* mutants we developed. In our study, molecular descriptors belong to MOE 10 data base were calculated for *C*-terminal Mad2 native and mutants (residues 190–205) and evaluated for their significant fluctuations. For a better interpretation of the results in the light of the SAR study, we selected twelve Mad2 mutants which adopt both open and closed configurations and interact with Cdc20 (Figure 2a–d).

Some molecular descriptors of Mad2 mutants show significant fluctuations (water accessible surface area induced by polar atoms and hydrophobic, dipole moment) while other descriptors have an insignificant fluctuation (van der Walls energy). Due to the fact that absolute numeric values of molecular descriptors are high, in order to better emphasize the variation of different parameters for various mutants, we calculated the absolute difference between descriptors values calculated for Mad2 native protein and similar descriptors calculated for mutants. The count of rigid and rotatable bonds and subdivided van der Waals surface areas (vsa_acc, vsa_pol, vsa_hyd) showed no fluctuations and the electric dipole moment recorded just an insignificant variation.

### 2.2. Discussion

#### 2.2.1. Power of QSAR Model to Predict of Mad2 Native and Its Mutants Binding against Cdc20

Our results generally support a good correlation between experimental and predicted Mad2 features for all training and testing molecules in the case of each QSAR model (residual values for training and test sets are comprised between 0.00 and 1.01). Concerning the interaction of Mad2 native and mutants with Cdc20, our results are in agreement with experimental results [10], indicating a good correlation between experimental and predicted biological activities of Mad2 native and mutants forms, the range of residual values being from 0.00 (Mad2 R133A/V163A) to −0.97 (Mad2 R133A/T12A) in QSAR model 2 and from 0.01 (Mad2 R133A/F151A) to 1.01 (Mad2 R133A/T12A) in QSAR model 1 (Table 2).

**Figure 3:**
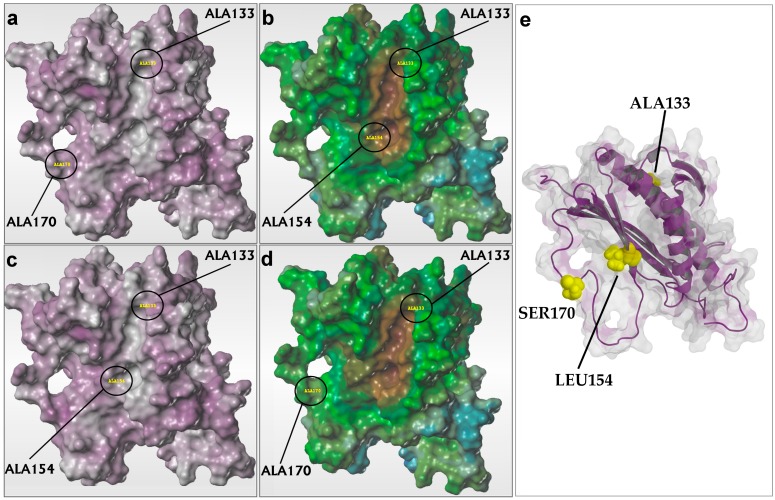
(**a**,**c**) The distribution of hydrogen bond acceptor (purple) and donor (grey) features on the water accessible surface area of Mad2 R133A/S170A (pCR_exp_ = 4.31) (**a**) and Mad2 R133A/L154A (pCR_exp_ = 4.37) (**c**); (**b**,**d**) The distribution of hydrophobic (brown) and hydrophilic (green) features on the water accessible surface area of Mad2 R133A/L154A (p*K*_d(Cdc20)exp_ = 5.91) (**b**) and Mad2 R133A/S170A (p*K*_d(Cdc20)exp_ = 7.09) (**d**); and (**e**) The backbone from the template structure used for modeling Mad2 mutants (Protein Data Bank code: 1S2H [17]) is represented as a purple ribbon with the same orientation as the models of Mad2 mutants presented in (**a**–**d**). The residues that present substitutions are represented with yellow spheres and are labeled accordingly.

Here we performed a detailed analysis of Mad2 molecular descriptors contribution at interaction with Cdc20. It is important to emphasize a positive contribution of structure-derived descriptors like van der Waals energy, water accessible surface areas generated by hydrophobic atoms and count of rotatable bonds and a negative contribution of the count of rigid bonds at binding of Mad2 to Cdc20. This is confirmed by the robustness of QSAR model 2 when the count of rotatable bonds was added as critical molecular descriptors at p*K*_dCdc20_ (Table 2). Figure 3b,d graphically support these observations presenting the distribution of hydrophobic properties on water accessible surface areas of two Mad2 mutants with low and respectively high values of experimental p*K*_dCdc20_: R133A/L154A (p*K*_dCdc20_ = 5.91) and R133A/S170A (p*K*_dCdc20_ = 7.09). The location of substituted residues in Mad2 structure is presented in Figure 3e. Figure 3d clearly indicates a larger distribution of hydrophobic area (brown) on Mad2 R133A/S170A compared to the distribution of the corresponding feature on Mad2 R133A/L154A (Figure 3b). Instead, by comparing the same figures, we noticed that the distributions of neutral (blue) and hydrophilic (green) areas are represented on both surface areas in an identical manner.

For a number of Mad2 mutants (Mad2 R133A/P164A; Mad2 R133A/T187A and Mad2 R133A/K192A) it was not possible to experimentally detect [10] their binding to Cdc20, even if it was confirmed that these mutants adopted both open and closed configurations and that they are able to interact with Cdc20. We extended the prediction power of QSAR models 1 and 2 and we calculated the p*K*_(dCdc20)pred_ for these mutants. Our results show that the p*K*_(dCdc20)pred_ for Mad2 mutants mentioned above, obtained by QSAR models 1 and 2 are included in the range of both experimental and predicted Mad2 mutants mentioned in Table 2 (Mad2 R133A/P164A (6.53/6.62), Mad2 R133A/T187A (6.60/6.69) and Mad2 R133A/K192A (6.53/6.53)). Based on our results, and on the experimental data [10,13] that proved the ability of these Mad2 mutants to interact with Cdc20 in a similar manner to Mad2 native, we suggest that QSAR models 1 and 2 are able to predict with sufficient accuracy the affinity of Mad2 mutants for Cdc20.

In addition, we predicted the binding affinity of Mad2 L13A at Cdc20 applying the statistical equations developed by QSAR models 1 and 2. Our computational results show that the predicted p*K*_(dCdc20)pred_ Mad2 L13A is very similar to the experimental p*K*_dCdc20_ for native Mad2 (p*K*_d(Cdc20)pred_ = 7.01) and close to the experimental p*K*_dCdc20_ of the most active protein of the series: Mad2 A133/A170 (p*K*_(dCdc20)exp_ = 7.09). We mentioned that our results are in good agreement with experimental data [10] showing that Mad2 13A mutants retained their ability to bind to Cdc20, and in addition, our results support the experimental observation that the present C-Mad2 A13 is the more active species of Mad2 for Cdc20 binding [10].

In QSAR model 1, the best fitting between experimental and predicted binding of Mad2 at Cdc20 was recorded for Mad2 R133A/F151A (residual value = 0.01), Mad2 R133A/L154A (residual value = −0.02) and, very importantly, for Mad2 native (residual value = 0.02). In QSAR model 2, the Partial Least Squares (PLS) regression resulted in a satisfactory predicted activity of Mad2 R133A/V163A (residual value = 0.00), Mad2 R133A/D158A (residual value = −0.02) and Mad2 native (residual value = −0.05). The most accurate predictions (residual value less than 0.2) were consistently achieved for almost all Mad2 structures indicating a good reliability of our QSAR models. Comparative analysis of the prediction power of QSAR models 1 and 2, expressed by the values of statistical parameters and also by the range of residual values, shows that the predictive power of QSAR model 1 is lower than that of QSAR model 2 (Table 2).

#### 2.2.2. Power of the QSAR Model to Predict Mad2 Native and Its Mutants in O-Mad2–C-Mad2 Interconversion Rate

Structural studies [4,10] mentioned the critical importance of the conformational changes of Mad2 native and mutants, namely the interconversion from inactive (O-Mad2) to active (C-Mad2). For the first time, by QSAR model 3, we predict the interconversion rate, even if the QSAR method usually considers biological activity as a dependent variable. Concerning the prediction power of QSAR model 3, we noticed that statistical parameters are satisfactory (Table 1), which gives us the possibility to predict the O–C-Mad2 interconversion rate and also to correlate it with experimental pCR_exp_. In QSAR model 3, the PLS regression leads to a generally good correlation between both types of interconversion rates (experimental and predicted); the best correlation being obtained for Mad2 R133A (residual value = 0.00); Mad2 R133A/K200A (residual value = −0.01) or Mad2 R133A/V163A (residual value = −0.02).

In the case of QSAR model 3 we noticed the positive contribution of protein dipole and hydrophobic moments and also van der Waals surface induced by polar atoms and a negative contribution of van der Waals surface induced by hydrogen bond acceptor atoms. A graphical illustration of these observations is in Figure 3a,c presenting the distribution of hydrogen bond acceptor/donor on water accessible surface areas of Mad2 R133A/L154A (pCR_exp_ = 4.37) and Mad2 R133A/S170A (pCR_exp_ = 4.31). We note the similar distributions of acceptor (grey)/donor (violet) features in the case of very close values of conversion rate of open–closed conformation of Mad2 mutants.

Previous experimental studies [10,13] showed that Mad2 native and Mad2 R133A/K192A are able to adopt both open–closed configurations, but the interconversion rates of these mutants were not detected. We predicted the open-closed configurations conversion rates for these Mad2 structures (Mad2 native, pCR_pred_ = 5.67 and Mad2 R133A/K192A pCR_pred_ = 3.30) (Table 2, in italics). In agreement with experimental observations [10] the predicted interconversion rates for Mad2 native and Mad2 R133A/K192A are included in the experimental range of pCR_exp_ and we suggest that these values are correctly predicted.

#### 2.2.3. Power of QSAR Model to Predict Mad2 *de Novo* Mutants Binding to Cdc20

The simultaneous substitutions of bulky amino acids like arginine 133 and phenylalanine 186 in double mutants Mad2R133A/F186A and in point mutant Mad2 F186A with small amino acids like alanine were experimentally demonstrated [10] to abolish Mad2 interaction with Cdc20 and conversion from open to closed configuration.

We present here a number of nine *de novo* Mad2 mutants with substitution in positions 133 and 186 (Table 2) for which the predicted mutant binding affinities against Cdc20 were compared with the experimental values of p*K*_(dCdc20)exp_ detected by experimental studies [10,15]. By applying QSAR models 1 and 2 and using experimental data [10,15], we suggested that when arginine and phenylalanine are substituted simultaneously or individually with small and less hydrophobic residues like alanine, the interaction of Mad2 mutants with Cdc20 is abolished (Mad2 F186A, p*K*_(dCdc20)pred_ = 5.77/5.96 and Mad2 R133A/F186A p*K*_(dCdc20)pred_ = 5.34/5.44). We made a similar observation in the case of simultaneous substitutions of arginine and phenylalanine with small and less hydrophobic residues (alanine, serine) and with a polar residue (asparagine) (Table 2). Instead, when simultaneous substitution with arginine 133 and phenylalanine 186 is performed with alanine and hydrophobic residues methionine, the predicted affinity of Mad2 R133A/F186M is close to experimental affinity of Mad2 in the native form (Mad2 R133A/F186M, p*K*_(dCdc20)pred_ = 7.11/7.24). A similar observation may be made when a single substitution in Mad2 F186 is performed with bulky and very hydrophobic residues like methionine and tryptophan. In this case, predicted p*K*_(dCdc20)pred_ of *de novo* Mad2 mutants have slightly higher values in comparison to native Mad2.

Having in mind the positive contribution of van der Waals and water accessible surface areas induced by hydrophobic atoms and also of the count of rotatable bonds, we suggest that the validity of QSAR models 1 and 2 is also reinforced by the results obtained in the case of *de novo* Mad2 mutants. All these observations allow us to suggest that overexpresion of Mad2 R133A/F186M and Mad2 F186M/W may not induce chromosomal instability but of course these proposals are necessary to be sustained also by *in vivo* studies. Study of theoretical chromosomal stability induced by these mutants could be extended and could represent new opportunities for pharmachogemonic studies.

An exception in our study is represented by Mad2R133A/F186T, when the presence of a less hydrophobic residue induced an experimental p*K*_dCdc20_ in comparable range with the p*K*_dCdc20_ values observed for mutants from Table 3.

Validity of QSAR model 3 was extended at *de novo* Mad2 mutants. We suggested that the predicted interconversion rate of Mad2 mutants: Mad2 R133A/F186M (pCR_pred_ = 4.36), Mad2 R133A/F186S (pCR_pred_ = 4.96), Mad2 R133F/F186T (pCR_pred_ = 4.97) and Mad2 R133A/F186N (pCR_pred_ = 5.26) may be included into the range of values that comprises the pCR values detected experimentally for the Mad2 mutants presented in Table 3. We can explain these results by the observation that these mutants presented the values for molecular descriptors with positive contribution at an interconversion rate (hydrophobic and dipole moments and also van der Waals area induces by polar atoms) close to the values for the correspondent molecular descriptors of Mad2 mutants presented in Table 3.

We have to mention that the lack of experimental data on *de novo* Mad2 mutants and native interactions with specific ligands Mad1 and Cdc20 imposes significant limitations on the impact of our study.

#### 2.2.4. SAR Analysis of Mad2 Native and Mutants at *C*-Terminal Domain Residues

The analysis of the fluctuation of molecular descriptors calculated for *C*-terminal Mad2 native and 12 mutants selected so that they present unmodified *C*-terminal domain (Figure 2a–d) showed that: (i) Water accessible surface area induced by hydrophobic atoms relative to Mad2 native varies significantly ranging from 8.25 Å^2^ (Mad2 R133A/D158A; Mad2 R133A/L154A) to 13.13 Å^2^ (Mad2 R133A); (ii) relative to native Mad2, the van der Waals energies show small variations ranging from 3.53 kcal/mol (Mad2 R133A/E179A) to 3.83 kcal/mol (Mad2 R133A/F151A); and (iii) in agreement with our expectations, the van der Waals surface area induced by hydrophobic atoms and the count of rotatable and rigid bonds remained unchanged.

Concerning the results on the fluctuation of molecular descriptors critical for the interconversion rate between open and closed conformations of Mad2 native and mutants mentioned before, the hydrophobic and dipole moment recorded significant fluctuation while the values of van der Waals surface areas induced by polar and hydrogen bond acceptor atoms were unchanged. We suggest that the fluctuation of the water accessible surface area, van der Waals energy and hydrophobic moment calculated for mutants in Mad2 *C*-terminal domain may be used to identify specific amino acids substitutions that affect Mad2 affinity for Cdc20 or the rate of protein folding by changes in the steric and electronic features of Mad2.

Even though the biological processes in which Mad1–Mad2 and also Mad2–Cdc20 interactions are involved are very complex and difficult to replicate in preclinical studies, the extension of our study by *in vivo* analyses of these *de novo* mutants is crucial to obtain new knowledge about pharmacogenetics of cancer.

## 3. Experimental Section

### 3.1. Dataset for Analysis

We used a database of 24 Mad2 proteins (native and 23 mutants) compiled from the literature [10,15]. Protein properties were expressed as: (i) Mad2 binding affinity for Cdc20 (*K*_dCdc20_), obtained by isothermal titration calorimetry; and (ii) Mad2 folding expressed as open-closed structure conversion rate constants (CR_open–closed-Mad2_) measured by NMR at 30.8 °C. These properties were originally expressed in micromolar (i) and 10-5s-1 (ii) and were converted to p*K*_dCdc20_ values by considering log(1/*K*_dCdc20_), respectively to pCR by calculating log(1/CR). Some of these values were not applicable (NA), no binding was detected (NBD) or binding was not determined (ND) [10]. The dependent variables of QSAR models developed in this study were p*K*_dCdc20_ and pCR.

Here we perform a structural–functional analysis of double Mad2 mutants belonging to all five classes and moreover, we extended our QSAR study to several singe Mad2 mutants whose activity is well documented in experimental studies. Such a mutant is Mad2 L13A, for which it was experimentally demonstrated that the mutation selectively destabilizes the open conformation of Mad2, arrests the protein in the closed conformation and preserves its ability to bind to Cdc20 [10,16]. In addition, it was shown that Mad2 L13A mutant and Mad2 native inhibited APC/C-Cdc20 in a similar manner [10]. In the present study, we also predict the behavior of Mad2 specific mutants that abolish Mad2 stability: F186A and R133A/F186A. Experiments showed that both mutants adopt only the open conformation and that the mutations altered the integrity of the protein, leading to the failure of Mad2 to interact with Cdc20. The Mad2 mutants included in this study were selected according to the following criteria: (i) The level of observed changes in Mad2 function (e.g., a correctly folded open-closed Mad2 conformation leading to appropriate interaction with Cdc20, an incorrectly folded Mad2 leading to Mad2 open conformation or on the contrary, a correct folding but an incapacity of Mad2 to interact with Cdc20); (ii) non-conservative mutations; and (iii) wide variability of values of Mad2 binding affinity to Cdc20.

### 3.2. Rational Design of de Novo Mad2 F186 and Mad2 R133/F186 Mutants with Possible Non-CIN Functions

An important objective of our study was to predict the function of possible *de novo* Mad2 mutants in positive or negative correlation with CIN. Based on experimental data [10,13] indicating that Mad2 F186A and Mad2 R133A/F186A substitutions affect protein integrity and lead to Mad2 inability to interact with Cdc20, we established nine *de novo* Mad2 mutants by rational-design following the most susceptible substitutions at residues R133 and F186.

Our computational mutagenesis strategy was based on several rules: (i) Variation of hydrophobic contacts by introducing hydrophobic amino acids like methionine and tryptophan and also less hydrophobic amino acids like serine, alanine, threonine; (ii) we changed the polar contacts by introducing polar amino acids, e.g., asparagine and mild polar amino acids, e.g., threonine and serine; and (iii) the molecular surface descriptors were changed by substitution with small amino acids, e.g., alanine, serine or bulky aminoacids, e.g., tryptophan. Thus, we introduced mutations as single substitutions: F186M, F186S, F186T, F186W and F186N and double substitutions: R133A/F186M, R133A/F186S, R133A/F186T, R133A/F186W, R133A/F186N and aimed to predict if these *de novo* Mad2 mutations induced chromosome instability by changing the values of molecular descriptors in comparison to Mad2 native and Mad2 mutants R133A/F186A and F186A.

### 3.3. Modeling of Native and Mutant Mad2 Proteins and Their Minimum Energy Calculation Strategy

Molecular modeling of the Mad2 native and mutants monomers presented in Table 2 and also of the *de novo* mutants proposed by rational-design, was performed using the Biopolymer module from Sybyl 7 software package (www.tripos.com) [28] using as template the X-ray structure of Mad2 mutant (1S2H PDB) [17]. The conformation with minimum potential energy of the Mad2 proteins was established using the conjugate gradient method routine in Sybyl 7, with Kollman force-field [29]. After energy minimization, Kollman partial charges [30] were loaded on the chemical structures from the Sybyl 7 dictionary. During energy minimization, free movements of the substituted amino acids were allowed.

### 3.4. QSAR Methodology

#### 3.4.1. Descriptors Calculations

Three dimensional structures of Mad2 native and mutants were uploaded in MOE 10 software [31] and 2D and internal 3D molecular features included into MOE 10 database were calculated. In the end those molecular features that follow the rules to avoid redundancy and chance correlation were selected, but were statistically relevant in order to allow an accurate validation of QSAR models [32]. The set of descriptors lead to the most statistic significant QSAR models which were based on the following combinations of descriptors:

QSAR model 1: p*K*_dCdc20_ = constant + *c*_1_ × EvdW + *c*_2_ × vsa_hyd + *c*_3_ × ASA_hyd + *c*_4_ × b_rigid_ (1)

where constant = −200.35, *c*_1_ = +0.038, *c*_2_ = +0.005, *c*_3_ = + 0.011, *c*_4_ = −0.040;

QSAR model 2: p*K*_dCdc20_ = constant + *c*_5_ × EvdW + *c*_6_ × vsa_hyd + *c*_7_ × ASA_hyd + *c*_8_ × b_rigid_ + *c*_9_ × b_rot_ (2)

where constant = −223.720, *c*_5_ = +0.036, *c*_6_ = +0.005, *c*_7_ = +0.012, *c*_8_ = −0.036. *c*_9_ = +0.030.

E-vdW represents the van der Waals energy as component of potential energy, vsa_hyd and ASA_hyd are considered as an approximation of the sum of van der Waals and water accessible surface areas of all hydrophobic [33]; b_rigid_ and b_rot_ are counts of rigid and rotatable bonds from proteins, QSAR model 3:

pCR = constant + *c*_10_ × vsa_pol + *c*_11_ × vsa_acc + *c*_12_ × M_dipole + *c*_13_ × M_hyd (3)

where constant = −89.114, *c*_10_ = +0.034, *c*_11_ = −0.035, *c*_12_ = +0.003, *c*_13_ = +0.005, M_hyd and M_dipole represent hydrophobic and electronic dipole moments [34] and vsa_pol and vsa_acc are considered as approximation of the sum of the van der Waals surface areas of all polar and hydrogen bond acceptor atoms [33].

Protein hydrophobic moment is a very important descriptor, especially when conformational changes are of interest and it is considered as a sum of the product between hydrophobicity of each amino acid and their distance *d_i_* between protein centroid and the centroid of residue *i* in space [34]. Protein binding occurs through interactions at the molecular surface described through van der Waals and/or solvent accessible surface areas. The protein molecular surface area determines various important properties with significant implications in protein–protein interactions or protein folding; therefore an accurate description of protein surface is crucial for understanding molecular recognition. Initial methods described proteins solvent accessible surface area as the surface “probed” by the center of a water probe sphere with a radius of 1.4 Å as it rolls over the van der Waals surface of the molecule, while a polyhedral representation is used for each atom in calculating the surface area. Since proteins contain huge number of hydrophobic and polar atoms, each of them with acceptor/donor of hydrogen bonds features, the above-mentioned limitations of computational methods performed on the global proteins surface [35] apply here. Taking this fact into account, we considered that the prediction accuracy of protein-protein interactions could be significantly improved if we performed an individual calculation on subdivided molecular surfaces segregated by types of atoms.

Based on experimental data indicating that the *C*-terminal domain is critical for Mad2 specific interaction with its partners Mad1 and Cdc20, the above mentioned molecular descriptors were calculated in the *C*-terminal domain (residues 190–205) for twelve Mad2 mutants (Figure 2). To deepen our understanding of Mad2 mutants, the mechanism of action during specific interactions, we selected those mutants with significantly different binding affinities against Cdc20 and whose amino acids sequence is unaltered in the *C*-terminal domain, but all substitutions are within domain 1–189.

#### 3.4.2. Chemometric Analyses

QSAR principles state that a reliable equation for structure activity relationship should possess good correlation coefficients (*q*^2^ (cross-validated *r*^2^) and fitted correlation *r*^2^), a low standard error of estimate prediction and the least possible number of variables [32]. Therefore in our study the validation criteria were *q*^2^ higher than 0.50, *r*^2^ higher than 0.80 and optimum number of principal components. The regression analysis was performed using the PLS algorithm within MOE 10 software [31]. The number of principal components (PCA) equal to 4 was chosen to achieve optimum values for statistical parameters *q*^2^ and *r*^2^, which were evaluated by applying the cross-validation and respectively non-cross-validated procedures available in MOE 10 software. In QSAR models 1 and 2, insignificant differences in values of the statistical parameters were recorded for PCA = 5 (*q*^2^ = 0.53, *r*^2^ = 0.83) and PCA = 6 (*q*^2^ = 0.51, *r*^2^ = 0.81). When PCA included more than five components, QSAR model 3 was non-valid due to serious over fitting (*q*^2^ = 0.60, *r*^2^ = 0.90). Also, contribution of PCA = 1–3 was very weak and may be irrelevant in all three QSAR models (*q*^2^ less than 0.30, *r*^2^ less than 0.70). Also Fisher test, RMSE (root mean square error) and cross-validated-RMSE were calculated [36].

#### 3.4.3. Training and Testing Sets

In the QSAR procedure applied to small molecules, the consistency of statistical models depends on the quality of both training and testing sets in terms of structural diversity and property value distribution. When the QSAR procedure is used to predict the features of Mad2 native and mutants, it is possible that the diversity and property value distribution of protein functions is in a small range and the validity of QSAR models fails. Besides, in our study, we had access to a small number of Mad2 mutants for which the experimental data are available. Despite these drawbacks, from the original data presented in Table 2, 17 Mad2 structures with K_dCdc20_ and 16 Mad2 structures with CR were randomly split into a training set of 12 proteins and a testing set of five proteins (QSAR models 1 and 2) respectively 11 compounds and a testing set of five compounds (QSAR model 3). Statistically significant QSAR models were generated, with testing sets containing different mutants, as follows:

QSAR models 1 and 2: R133A; R133A/L153A; R133A/D160A; R133A/Y156A; R133A/T12A;

QSAR model 3: R133A/L84A; R133A/I88A; R133A/F151A; R133A/S170A and R133A/W167A.

A statistical cluster analysis confirmed that the composition of both training and testing sets is representative for the whole data set (Figure 1a,b).

## 4. Conclusions

Molecular simulation techniques such as rational design of protein mutants and structural–enzymatic activity relations will continue to reveal important information about protein function or the implication of proteins in many cellular processes such as correct chromosome segregation (euploidy), but it is important to understand the limitations and challenges of these techniques. In the present study we considered a number of 26 Mad2 structures—native and mutants (16 already known to induce aneuploidy and nine proposed by us by computational mutagenesis). These mutants were included in three QSAR models used for the prediction of Mad2 affinity for its specific partner Cdc20 and also features like the interconversion rate between an open and closed configuration. In our study we determined that, among various structural descriptors considered, the steric (van der Waals area and water accessible surface area and their subdivisions) and also energetic van der Waals energy descriptors are more relevant for predicting the involvement of Mad2 native and mutants in genetic disorders and their mechanism of action. This is a prerequisite for the development of effective methods for early diagnosis and for possible treatment strategies.

In addition, we conclude that the evaluation of protein hydrophobic and dipole moments as well as van der Waals surface areas over all polar and hydrogen bond acceptor atoms, may be important for computational prediction of the Mad2 mutants role as inductor of chromosome instability.

We suggest that the molecular descriptors of native and mutants Mad2 evaluated here represent important resources for future computational studies focused on aneuploidy, provided that kinetic data about Mad1–Mad2 and/or Mad2–Cdc20 are available.

We are confident that in future, our study can be extended by *in vivo* techniques which are able to explore more precisely the *de novo* Mad2 mutants presented here.
